# Focused Neuro-Otological Review of Superficial Siderosis of the Central Nervous System

**DOI:** 10.3389/fneur.2018.00358

**Published:** 2018-05-28

**Authors:** Aran Yoo, Jonathan Jou, Jeffrey D. Klopfenstein, Jorge C. Kattah

**Affiliations:** ^1^University of Illinois College of Medicine, Peoria, IL, United States; ^2^Department of Neurosurgery, University of Illinois College of Medicine, Peoria, IL, United States; ^3^Department of Neurology, Illinois Neurologic Institute, University of Illinois College of Medicine, Peoria, IL, United States

**Keywords:** superficial siderosis, subarachnoid hemorrhage, neuro-otology, bilateral vestibulopathy, video head impulse

## Abstract

**Background:**

Infratentorial siderosis (iSS) is a progressive degenerative disorder targeting primarily the cerebellum and cranial nerve eighth; therefore, progressive ataxia and its neuro-otological findings are common. Toxicity from hemosiderin involves selectively vulnerable neurons and glia in these posterior fossa structures. Other neurologic findings may be present, though our focus relates to the cochlea-vestibular cerebellar involvement. Radiographic evidence of siderosis may be the result of recurrent, albeit covert bleeding in the subarachnoid space, or the consequence of an overt post-traumatic or aneurysmal subarachnoid hemorrhage (SAH). The radiographic iSS appearance is identical regardless of the SAH cause. A recent study provides compelling evidence to search and correct possible hemorrhage sources in the spinal canal. The removal of residual existing hemosiderin deposits that may potentially cause clinical symptoms remains as a major therapeutic challenge.

**Methods:**

We reviewed large data sources and identified salient papers that describe the pathogenesis, clinical and neurotologic manifestations, and the radiographic features of iSS.

**Results:**

The epidemiology of iSS is unknown. In a recent series, clinically evident iSS was associated with recurrent SAH; by contrast, in a follow-up period ranging from weeks up to 11 years after a monophasic episode of SAH, radiographic siderosis was clinically silent. However, the post-aneurysmal or post-trauma SAH sample size in this single study was small and their observation period relatively short; moreover, the burden of intraneuronal hemosiderin is likely greater with recurrent SAH. There are a few reports of late iSS, several decades after traumatic SAH. A recent report found subjective hearing loss in aneurysmal SAH individuals with radiographic siderosis. Only in recent years, it is safe to perform magnetic resonance imaging (MRI) in post-aneurysmal SAH, because of the introduction of titanium, MRI-compatible aneurysm clips.

**Conclusion:**

iSS can be associated with significant neurotologic and cerebellar morbidity; the recurrent SAH variant is frequently clinically symptomatic, has a shorter latency and greater neurotologic disability. In these cases, a thorough search and management of a covert source of bleeding may stop clinical progression. The frequency and clinical course of radiographic iSS after traumatic and post-aneurysmal SAH is largely unknown. Detection of radiographic iSS after trauma or aneurysm bleeding suggests that the slower clinical course could benefit from an effective intervention if it became available. The use of cochlear implants is a valid alternative with advanced hearing impairment.

## Introduction

Superficial siderosis (SS) of the central nervous system (SSCNS) is an uncommon neurodegenerative disorder. While two types of SSCNS exist, we focus on infratentorial superficial siderosis (iSS) and its neuro-ontological findings. iSS involves chronic bleeding into the subarachnoid (SA) space and hemosiderin damage along the auditory pathway, including the cochlear nerve axon, cochlea, cochlear nuclei, and auditory cortex. Hearing loss is the most common manifestation of SSCNS, beginning with involvement of high frequencies, and sometimes progressing to severe bilateral deafness. We review the efficacy of cochlear implants in iSS. Vestibulopathy is a less common manifestation of SSCNS; therefore, it is potentially be overlooked, especially in the setting of cerebellar ataxia. Concurrent presentations of ataxia and vestibular impairment should alert clinicians to possible diagnosis of SSCNS. In this review, we provide an overview of the pathophysiology and methods of diagnosis of SSCNS vestibulopathy.

The pathogenesis of SS involves chronic neuronal damage *via* hemosiderin oxidation and deposition onto the leptomeningeal surfaces of the brain, cranial nerves, and spinal cord (1). There are two broad anatomically based iSS categories. (1) *Cortical siderosis* (*cSS*) that affects the supratentorial compartment and is most commonly associated with cerebral amyloid angiopathy (2). We will not discuss this variant in this review, it is possible that it relates to recurrent small bleeding events and bears a pathogenic link with the recurrent subarachnoid hemorrhage (SAH) variant of iSS. (2) *Infratentorial superficial siderosis* (iSS) that involves hemosiderin deposition onto the brainstem, cerebellum, cranial nerves, and spinal cord. ISS is commonly associated with the cardinal features of SSCNS: sensorineural hearing loss, cerebellar ataxia, and pyramidal tract signs (1). Although iSS is an infrequent cause of symmetric cochleovestibular loss, it is often the first iSS symptom and magnetic resonance imaging (MRI) represents the key diagnostic test. In this review, iSS will be our primary focus. Formerly, the diagnostic emphasis was the identification of an intracranial source of bleeding, recent advances on the pathogenesis of iSS identified indolent spinal canal lesions as a possible bleeding source. Our intent in this review is to summarize the literature in this topic as it applies to patients with slowly progressive bilateral sensorineural hearing loss and vestibulopathy.

## Methods

A search of publications on databases PubMed, Cochrane Library, and CINAHL with the inclusion of the term “superficial siderosis” and exclusion of the terms “cortical,” “CAA,” and “amyloid” was performed to review infratentorial SSCNS. We reviewed abstracts of articles read in entirety if found to be relevant. We included only articles written in the English language. In addition, we reviewed a handful of papers reporting the effect of treatment. We did not include a few single case reports, because in our view they would not add further information in the understanding of this entity. For this reason, this is a focused though comprehensive literature review of iSS.

## Pathophysiology and Diagnosis

**Figure 1:**
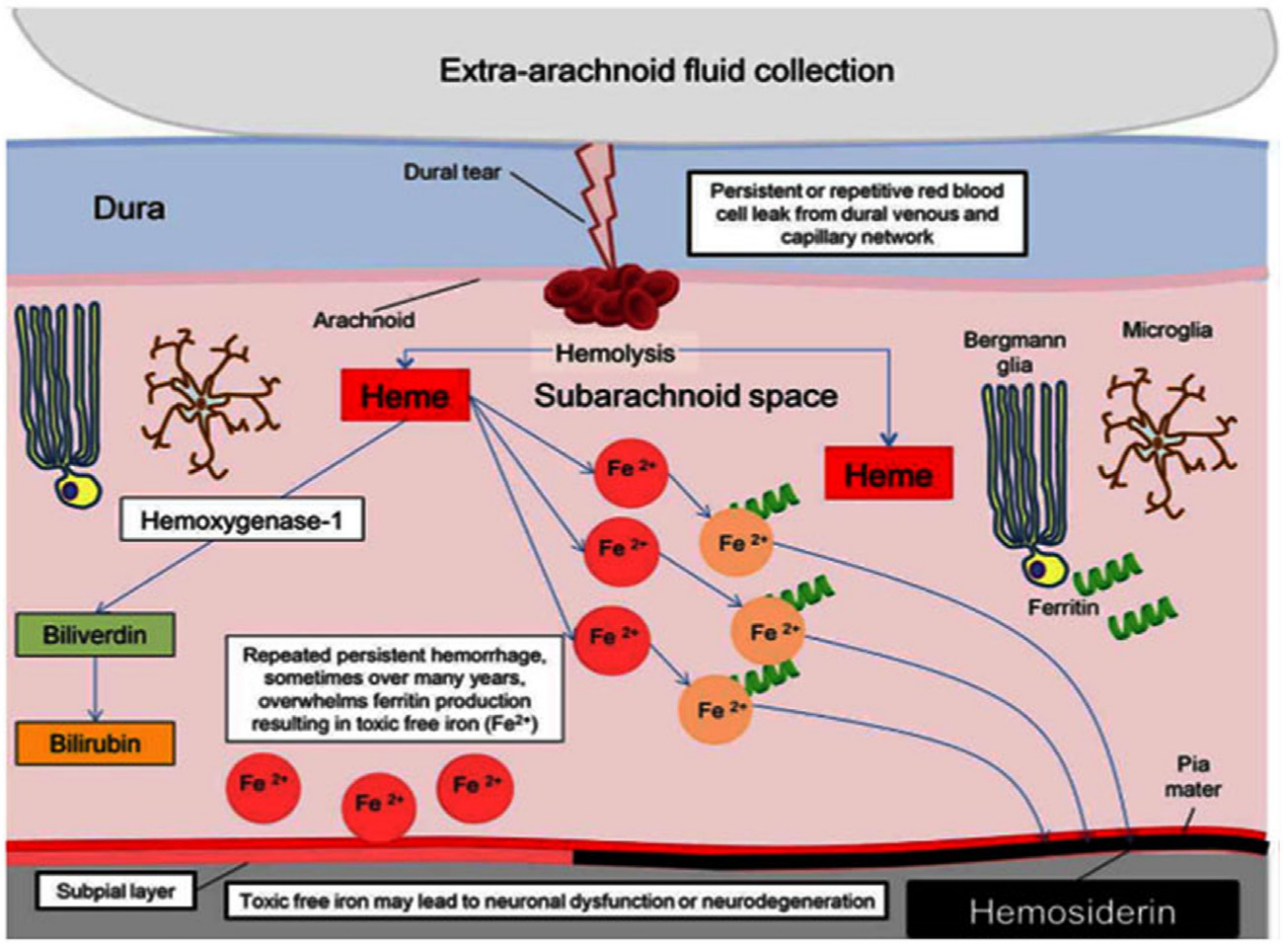
Pathophysiology of hemosiderin damage in classical infratentorial superficial siderosis [from Wilson et al. (4)].

Since Iwanowski and Olszewski’s experiment with injection of blood into the SA space, repeat hemorrhage into the cerebral spinal fluid (CSF) is the accepted mechanism of iSS (3). Microscopically, microglia and Bergmann glia digest deposited red blood cells and accumulate hemosiderin intracellularly. Glial cells eventually become overwhelmed and release hemoxygenase-1 and ferritin. Hemoxygenase-1 is a source of free iron that leads to oxidative stress, causing gliosis, and neuronal death. When ferritin binds free iron, hemosiderin forms and its deposition is radiographically detectable. Figure 1 from a recent review illustrates the current theoretical process (4).

Damage occurs commonly in the brainstem, cerebellum, ventricles, and vestibulocochlear system due to the richness of glial cells in this area (5, 6). Concerning iSS there are two types; the first relates to repeat episodes of bleeding and the second relates to a monophasic aneurysmal or traumatic SAH or intracerebral hemorrhage (ICH). Various etiologies for chronic iSS were reported in the past, including principally disruption of meningeal integrity (dura mater tears) and vascular abnormalities. Examples including root avulsion, meningoceles, pseudomeningoceles, and tumors myxopapillary ependymomas are particularly common (1). Interestingly, there is significant variance in reported latency periods between initiation of damage causing chronic hemorrhage and iSS first symptoms. While it takes roughly 6 months for hemosiderin to deposit after weekly autologous red cell injection in animal models (7), human patients experience an asymptomatic period anywhere from 2 to 51 years (8). The amount of bleeding, and whether it is monophasic, or recurrent, explains the latency and diversity of iSS clinical presentations.

**Figure 2:**
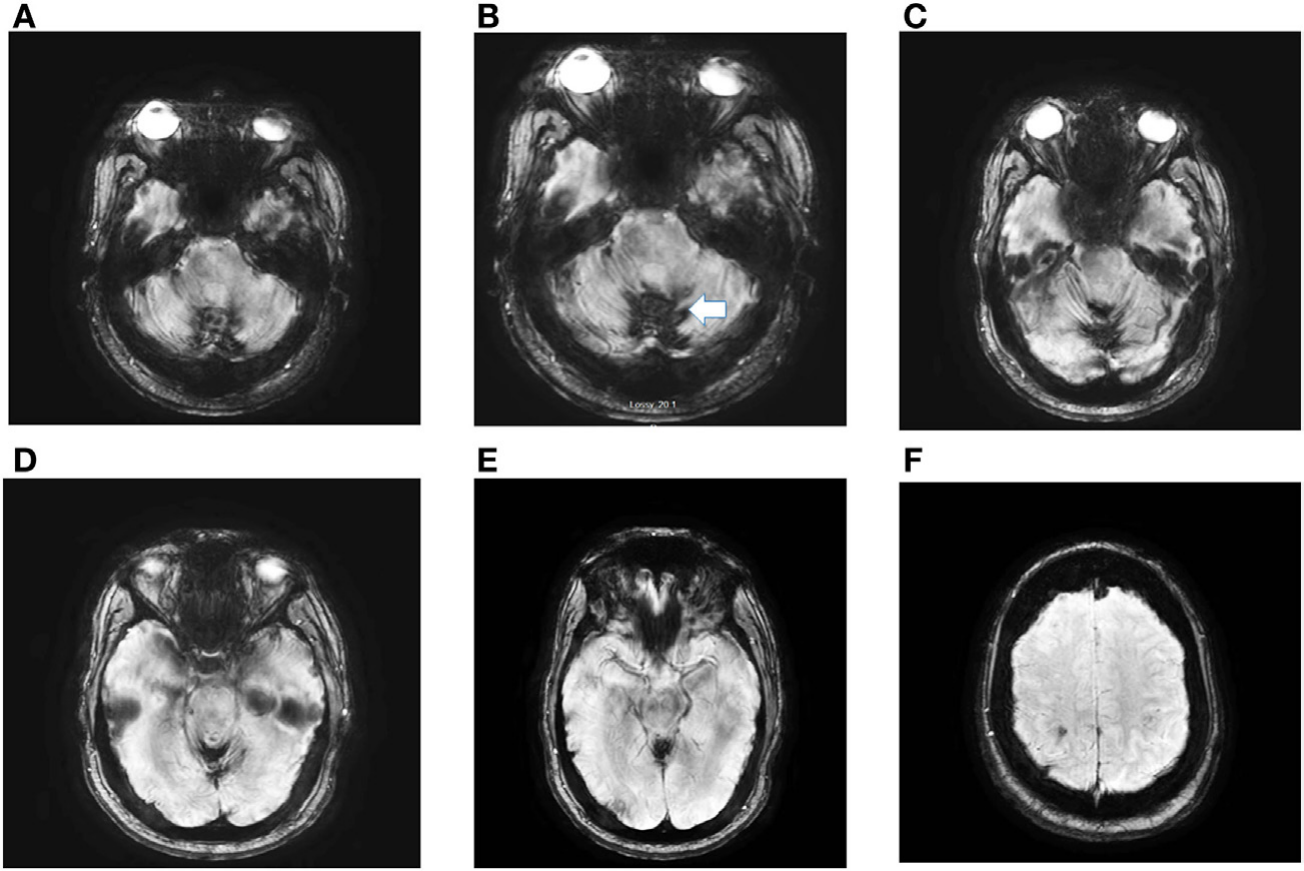
Axial susceptibility weighed (SWI) magnetic resonance imaging (MRI) obtained in a 56-year-old man with post-traumatic iSS. The MRI, detected extensive hemosiderin deposition in the cerebellar folia (iSS) **(A–E)**. Panel **(F)** shows minimal cSS. He had paroxysmal positional vertigo involving the right posterior canal, bilateral, symmetric high-frequency-hearing loss, decreased vertical angular VOR gain in the vertical canals, except for normal right posterior gain. He has significant gait and limb ataxia. At age 4, he had a head injury during a MVA with prolonged coma.

Technological advancements have improved the ability to diagnose iSS significantly so that diagnosis is no longer reliant on post-mortem evaluations. Hemosiderin coating is demonstrated on MRI, especially with gradient echo (GRE) and T2-weighted imaging, as a rim of hypointensity in the cerebellar hemispheres, brainstem, and cerebral hemispheres (9) (Figure 2). The weakness of MRI diagnosis, however, relates to the unknown relationship between radiographic findings and symptomatic disease. In a study of 13 patients with iSS diagnosed with MRI findings, only 15% had clinical symptoms (10). Imaging, however, will continue to be the gold standard test in the evaluation of SSCNS patients. In their seminal paper by Wilson et al., a diagnostic algorithm for iSS includes various potential imaging techniques, including cranial and spinal MRI, computed tomographic (CT) myelography, CT angiography, magnetic resonance angiography, and intra-arterial digital subtraction angiography; in order to find the underlying cause of iSS (11). These authors performed a prospective and retrospective review of 65 patients and divided the patient population into two groups: those with the classic clinical picture (termed “type 1” iSS) and those without (termed “type 2” iSS). Type 1 iSS patients had at least one of the “classical” and slowly progressive symptoms of SSCNS, while type 2 iSS did not. Radiographically, iSS1 and iSS2 are similar. With this classification, Wilson et al. were able to use various imaging techniques to find a potential etiology for 94% of type 1 iSS patients. Clinicians must be particularly aware of an indolent spinal cord lesion. Intraspinal CSF collection, nerve root cysts, posterior fossa cysts represent frequent imaging findings (12). Spinal MRI and CT myelography had the most successful rates of discovering potential underlying etiologies of iSS (11, 13). In the case of high flow leaks, ultrafast dynamic-CT myelography may more accurately localize the leak site (14). Smaller leaks may be hard to detect with any modality, however (15). Other more sensitive techniques may develop with time; Arishima et al. were able to detect a dural tear with endoscopic methods (15). Notably, although these etiologies require further investigation to strengthen the diagnostic algorithm suggested by Wilson et al., the authors’ study provides a significant advancement and improvement SSCNS diagnosis and management, as elimination of the source of recurrent bleeding is a key management factor. Before this study, most etiologies of iSS cases were often idiopathic (6, 12).

## Etiology

There are various underlying etiologies for chronic SAH. A comprehensive review of the literature was first performed by Fearnley et al. in 1995, and much of the understanding of the initial epidemiology of SSCNS relied on this study. When a source of bleeding was reported, it involved predominantly dural pathology (47%), including a tumor in 35% (e.g., myxopapillary ependymoma), CSF cavity lesion in 29% (e.g., hemispherectomy), cervical root lesion in 18% (e.g., epidural cyst and avulsion), and vascular abnormality (e.g., arteriovenous malformation) in 18% (1). It is likely that the SSCNS cases with underlying dural pathologies are type 1 iSS (11). In iSS, hearing loss and ataxia are the dominant symptoms, by contrast, cSS impaired cognition, and seizures are the main manifestations.

Type 2 iSS associated with aneurysmal or traumatic SAH is a theoretically preventable etiology. The most common mechanism of these SAH are aneurysmal rupture associated at times with ICH (11, 16). In the case of aneurysmal SAH, monophasic hemorrhage leads to radiographic iSS, with radiographic findings associated with both supra- and infratentorial SSCNS. Possibly, because of the long latency between SAH and the first symptoms of SSCNS, in addition to lack of prolonged monitoring for iSS, the potential evolution from radiographic iSS to clinically symptomatic iSS is unknown. Notably, only recently have titanium aneurysmal clips and intravascular techniques enabled post-intervention MRI testing (17). One study analyzed the risk of SSCNS from a single aneurysmal SAH episode. At a single-institution, 72 patients with history of definitive diagnosis and treatment for a single episode of aneurysmal SAH were assessed with MRI, and 54% of these patients were found to have radiologic evidence of SS (average follow-up time was 47.4 months). Unexpectedly, out of the 88.9% of study infratentorial SAH patients, only 21.9% of patients developed iSS. Another study reported subjective hearing impairment in 20% of patients after aneurysmal SAH (18). Unfortunately, this study lacked formal audiometry evaluation.

Lummel et al. suggests that more complex pathophysiology may contribute to the classical symptoms of SSCNS, especially since *symptomatic* iSS after a single episode of SAH is rare (19). The latency for symptomatic iSS following monophasic SAH may be longer than that of iSS related to recurrent SAH. In fact, radiographic iSS occurs shortly after a single aneurysmal or traumatic iSS (19, 20), what is unknown, however, is if radiographic siderosis will evolve to a classical iSS picture (11). Moreover, it is unclear if the glia’s ability to handle intermittent though frequent exposure to hemosiderin is different from a single though sizable SAH; this question is interesting and important to address, as it also applies to the hemispheric variant of SS in the context of CAA.

## Signs and Symptoms

In the early review of SSCNS, Fearnley et al. reviewed clinical features of SSCNS. Authors widely agree that sensorineural hearing loss, cerebellar ataxia, and pyramidal signs are cardinal features, and according to Fearnley et al. were present in 95, 88 and 76% of patients in 63 cases, respectively (1). Other symptoms include urinary pathology, headaches, anosmia, ageusia, diplopia, bowel problems, cranial nerve palsies, memory deterioration, personality changes, and nystagmus (21). In a case report of one patient, a comprehensive neuropsychological assessment revealed impairment in a variety of cognitive functions (e.g., memory and executive function) (22). Interestingly, significant variance exists of reported latency periods between initiation of chronic hemorrhage and SSCNS symptoms. While it takes 6 months for hemosiderin to deposit after weekly autologous red cell injection in animal models (23), human patients experience an asymptomatic period anywhere from 2 to 51 years (8). Presumably, the degree of bleeding and monophasic versus intermittent recurrent bleeding results in the significant diversity of SSCNS presentations.

## Sensorineural Hearing Loss

Sensorineural hearing loss is the most common, and earliest, symptom experienced by SSCNS patients. According to a longitudinal single case report characterizing hearing loss in SSCNS showed over a 15-year period, showed prodromal tinnitus followed by sensorineural hearing loss and eventually total deafness (24). The hearing loss affected initially high frequencies and was initially asymmetric. Hearing loss in SSCNS can mimic presbyacusis (25); however, when matched against individuals of the same age and sex, SSCNS patients’ hearing deficit generally exceeds the degree expected from aging. In fact, 2,000 Hz is the most vulnerable frequency to fall outside of the normative range (26).

The pathophysiology of hearing loss may involve several potential areas of damage in the auditory pathways. One of the early-proposed mechanisms was retro-cochlear damage. Physiologic studies suggested selective damage according to the tonotopic organization of the cochlear nerve axons; this would explain why high frequency sounds are the first in SSCNS (17). Another mechanism includes cochlear damage; his possibility is supported by electrophysiologic parameters (compound action potentials and evidence of recruitment detected by pure tone audiometry) (17, 27). Importantly, temporal bone histopathology demonstrated hemosiderin damage to the organ of Corti, especially the spiral ganglion and the sensory cells (28). In a pathologic study, a patent cochlear aqueducts or internal auditory canals may promote circulation of hemosiderin-laden CSF within the cochlea (29). Moreover, this mechanism also applied to the experimental application of FeCl_3_ solution to the oval window of male rat ears. Spontaneous nystagmus appeared within 1 h and histopathological findings revealed partial loss of hair cells and atrophy of ampulla neurepithelium. In addition, affected ears had increased expression of markers of oxidative stress (30). Theoretically, other sites of involvement for audiological pathology in SSCNS include the cochlear nuclei within the floor of the fourth ventricle and the auditory cortex (25, 31, 32). The use of brainstem auditory evoked responses may be helpful in identifying precisely the auditory pathway lesion location (33). Primary loss of cochlear function (wave 1) with preserved brainstem auditory conduction will be a good predictor of cochlear implant success.

## Vestibulopathy

Vestibular dysfunction is a less common manifestation of SSCNS. Patients with SSCNS vestibulopathy present with dizziness often along with the common symptoms of progressive hearing loss and gait instability (8). Concurrent presentations of ataxia and vestibular impairment should alert clinicians to possible diagnosis of SSCNS (Table 1). The focused deficit of the eighth cranial nerve, and seen with the olfactory nerve, highlights the pathophysiology of SSCNS (37). The vulnerability of axonal damage results from the long distance it travels outside of the brain within the pontine cistern before it enters the internal acoustic canal. This area is additionally thought to have a larger pool of CSF along with greater flow, potentially delivering more iron into the area (6) The transition from central to peripheral myelin in the auditory nerve (Obersteiner-Redlich zone) is in this region; where central myelin is more susceptible to hemosiderin toxicity. The vestibulocochlear nerve also has a rich collection of microglia, which processes red blood cells into hemosiderin (23). As the microglia process extraneous blood, the nerve is exposed to more hemosiderin and reactive oxygen species (7).

**Table 1:** Causes of combined ataxia and vestibular loss (34–36).

Superficial Siderosis of the Central Nervous System
CANVAS: cerebellar ataxia, neuropathy and vestibular areflexia syndrome
Spinocerebellar ataxia (SCA) type 3 (Machado-Joseph disease) and others
Wernicke’s encephalopathy
Paraneoplastic syndromes
Mitochondrial syndromes
Friedreich’s ataxia
Vestibular schwannoma with cerebellar/brainstem compression
Anterior inferior cerebellar artery infarction
Vitamin B12 deficiency
Bovine spongiform encephalopathy

Clinicians should consider iSS1 in patients with progressive simultaneous cochleovestibular loss, particularly if they have additional signs of cerebellar dysfunction. Comprehensive auditory and vestibular testing will be confirmatory physiologic test, however, MRI is the gold standard and must include GRE, in addition, susceptibility weighted imaging (SWI) and susceptibility weighted angiography known by the acronym (SWAN) provide a precise identification of hemosiderin and establish the diagnosis (Figure 2).

Vestibulopathy is a SSCNS symptom/sign potentially overlooked, especially in the setting of cerebellar ataxia. A wide variety of diagnostic tools are used to assess vestibulopathy, including postural testing, electronystagmography, rotary chair tests, vestibular evoked myogenic potential testing, caloric test, and video head impulse test (vHIT) (23). Recently, the vHIT allows clinicians to assess early vestibular dysfunction due to suspected iSS1. vHIT can detect covert corrective saccades in vestibulopathy; it is more practical and sensitive than other traditional tests of vestibular function (8, 31). To our knowledge, only two case reports used vHIT for SSCNS evaluation; in these reports, the severity of vestibular loss (near vestibular areflexia) and the long latency period between the inciting events probably explains the low VOR gain (8). Multicenter trials may be able to recruit enough patients to explore and validate these monitoring tools.

## Treatment

Current recommendations for management of symptomatic SSCNS emphasize eliminating the mechanism of bleed. In a review of 23 cases of surgical treatment for SSCNS, 15 cases reported stabilization of patient condition and two cases reported progression of disease. Six cases reported clinical improvement, two of which subsequently deteriorated (38). Examples of surgical options include intradural exploration with subsequent embolization or bipolar coagulation of bleeding sources, ligation and resection of meningoceles or pseudomeningoceles, release of adhesions, resection of tumors, repair of dural tears, and lumbar drainage (14, 38).

In case reports, the results of intervention are difficult to tabulate for the following reasons: (1) the follow-up duration ranged from 1 month to 35 years; (2) the disease monitoring was usually performed simply with clinical assessment, and lacked objective findings (for example, absence of CSF analysis for xanthochromia); and (3) physiologic tests such as pure tone audiometry or vHIT were not utilized (39–43). Efficacy of surgical treatment was therefore difficult to assess within the current literature.

Aside from surgical options to treat symptomatic SSCNS, ideally removing any deposited hemosiderin is desirable. Several authors have explored lipid-soluble iron chelators, such as deferiprone, as potential treatment options with limited success. Moderate radiological improvement is with deferiprone (44); however, significant clinical improvement seems unlikely. Cummins et al. reported improved gait and hearing bilaterally after 4 months of deferiprone treatment; however, 6 months later, hearing deteriorated to pre-treatment levels (45). Kuo et al. found an improvement in Scale for the Assessment and Rating of Ataxia score by 2.5 points in one patient after 6 months (46). In a recent 2-year observational, study by Kessler et al. with data for 30 subjects, moderate subjective success was achieved: 40% of patients reported stabilization of hearing function and 30% stable or improved gait or coordination (47). Given limited success, the benefit of deferiprone may not outweigh its risks. One case of agranulocytosis with deferiprone has been reported (48), although no cases were found in the 30-case study (47).

Cochlear implants may lead to symptomatic improvement in sensorineural hearing loss associated with SSCNS. The success rate varies, possibly due to the variability in the location of sensorineural hearing pathology. Most authors only offer cochlear implants once hearing aids become inadequate. At this point, however, hearing loss may be significant. Trans-tympanic promontory electrical stimulation may be the gold standard method to define eligibility for cochlear implant candidates, especially in those patients with severe sensorineural hearing loss (6, 33, 49, 50). For example, in one case study, an audiological exam showed profound sensorineural hearing loss with preservation only of highly elevated low-frequency thresholds. Unaided speech discrimination scores were zero and evoked brainstem responses were absent at the highest stimulation levels. The authors proceeded with a left cochlear implant despite these unfavorable predictors based on an electrical promontory test that suggested functional integrity of the left eighth cranial nerve compared to the right. Brainstem auditory evoked responses may be helpful (33). Postoperatively, open-set speech perception improved compared to preoperative function (33).

Overall, cochlear implants have some benefit in SSCNS. In a systematic review of the literature, 47% of 15 individual cases demonstrated “clear sustained benefit” from cochlear implantation. The remaining cases showed absence or limited benefit (51). Other reviews have reported similar rates of success (52–54). From our review, the need for additional prospective studies to identify favorable factors that increase the success of cochlear implants is obvious.

## Conclusion

We provide a literature review of the important considerations in the pathogenesis of the sensorineural hearing loss/peripheral vestibulopathy and ataxia syndrome associated with SSCNS (iSS type 1). These observations may apply also to additional neurologic abnormalities such as cranial neuropathies, pyramidal tract dysfunction, and others. The MR imaging sequence must include SWI. We believe that radiographic (iSS type 2) evolution to clinically symptomatic iSS1 in aneurysmal/traumatic SAH is currently underestimated. The availability of titanium clips, enable future imaging monitoring of post SAH siderosis with a better appreciation of its frequency (55). The deficits may evolve over a long period and could be potentially prevented if an effective hemosiderin chelator became available and used in a timely fashion. We outline the diagnostic process for atraumatic siderosis in patients without aneurysmal SAH. We hypothesize that iSS2, may have a longer latency to symptomatic state; the incidence of symptomatic iSS in this population is unknown. Finally, the ideal management of “radiographic siderosis” includes the identification and removal of the source of bleeding in covert SAH, and in providing an effective siderosis blocking intervention for all cases, regardless of the etiology. As a final statement, to date, only one agent has shown a hint of promise, however, potential for high-rate of side effects may limit its potential use. Finally, there is high demand for such agent now that radiographic detection of iSS will likely increase.

## Author Contributions

AY, JJ, and JCK: writing of manuscript. JDK: critical revision of manuscript for intellectual content.

## Conflict of Interest Statement

JCK received research equipment ion loan form GN Otometrics in 2012, this equipment is no longer in use. The remaining authors have no disclosures. The reviewer GF and handling Editor declared their shared affiliation.
